# Molecular and Genetic Regulation of Crop Root System Architecture in Drought Resilience

**DOI:** 10.3390/plants15071048

**Published:** 2026-03-28

**Authors:** Yawen Wang, Kai Xu, Shoujun Chen, Siya Hang, Tiemei Li, Huaxiang Cheng, Lijun Luo, Liang Chen

**Affiliations:** 1 Shanghai Agrobiological Gene Center, Shanghai 201106, China; 2 College of Plant Sciences & Technology, Huazhong Agricultural University, Wuhan 430070, China; 3 Key Laboratory of Grain Crop Genetic Resources Evaluation and Utilization, Ministry of Agriculture and Rural Affairs, Shanghai 201106, China

**Keywords:** crop, drought resilience, genetic breeding, root system architecture

## Abstract

Drought, a major abiotic stressor affecting global agricultural productivity, significantly reduces crop yields and threatens food security worldwide. As the primary organ for perceiving soil moisture signals and absorbing water, the crop root system architecture plays a pivotal role in plant adaptation to drought conditions. With the development of high-throughput imaging technologies (i.e., 2D/3D image acquisition), high-throughput genotyping platforms, and gene-editing technologies, significant progress has been achieved in the characterization of root traits and the dissection of molecular genetic regulatory networks underlying these traits in crops. This review comprehensively synthesizes recent advances in the phenotypic characterization, underlying molecular regulatory networks, and functional roles of key root architectural traits, including the root length, angle, density, and root hair development, in enhancing drought resilience. Finally, we discuss the existing challenges in the current research and provide an outlook on the future trend of integrating multi-omics, high-throughput phenomics, and genome editing technologies to breed new drought-resistant crop varieties with ideal drought-resistant root architectures.

## 1. Introduction

Water serves as a critical limiting factor in agricultural production in water shortage regions, especially in Africa. Drought stress disrupts the delicate water balance in plants, suppresses photosynthesis, and perturbs physiological metabolism, ultimately causing substantial global declines in crop yield and quality [1]. In developing countries, a decline of 9–21% of the overall potential agricultural productivity was recorded as a result of drought and heat [2]. Escalating global climate change has amplified both the frequency and severity of drought events. It has been reported that the global reduction in rice production due to drought averages 18 million tons annually [3]. Annual global crop yield losses attributable to drought stress are greater than those caused by other abiotic stresses. Leveraging crop intrinsic genetic potential for enhanced drought resilience therefore constitutes a promising strategy against an urgent global challenge [4].

Crop adaptation to drought stress involves complex mechanisms at the morphological, physiological, and molecular levels. Among these, the root is the organ in direct contact with the soil, and its architecture and xylem vessel determine the efficiency of water acquisition. The xylem vessel traits serve as the “valve” regulating axial water transport efficiency [5]. Selected wheat varieties with narrow vessels exhibited significantly high yield compared with control varieties under drought field conditions [6]. Additionally, the root system architecture plays an important role in drought adaptation by regulating the spatial range and efficiency of water uptake [7]. Early studies proposed the concept of an “ideal root type” characterized as steep, cheap, and deep [8]. This root system configuration balances water acquisition efficiency, resource investment economy, and environmental adaptability, thereby helping to achieve stable crop yields under water-limited conditions. For example, longer roots with deep penetration can access water from deeper soil layers, vertically oriented roots with narrow angles reduce competition for shallow water, and dense lateral roots and root hairs enhance soil water absorption [9,10]. It is particularly pointed out here that when available water is typically located in shallow soil, deep root growth represents an ineffective investment. Therefore, the optimal root architecture should be selected based on the actual soil and moisture conditions in crop breeding [11]. Therefore, systematically elucidating the relationship between crop root morphological traits and drought resilience, and considering their synergistic effect with environmental conditions (i.e., soil, climate, rainfall patterns), along with the molecular regulatory mechanisms, holds significant theoretical and practical importance for improving crop drought resilience and ensuring food security.

Traditional breeding has often focused on aboveground traits, with insufficient attention paid to root systems “hidden underground” because of the difficulty in the investigation of root traits in the field. For decades, X-ray computed tomography (CT) and magnetic resonance imaging have been applied in non-destructive detection on crop roots [12]. In recent years, as the development of 2D/3D image acquisition through root scanning, coupled with advances in image analysis methods, significant progress has been made in studies of crop root phenomics. Combined with the development of genomics and molecular biology, a large number of quantitative trait loci (QTLs) and some genes/superior alleles controlling root traits have been identified in crops. The central roles of hormonal signaling networks, transcriptional regulatory modules in precisely modulating crop root morphology and response to drought, have been gradually revealed [13,14,15]. Field-based drought resilience evaluation conducted on genetic and breeding materials have further assessed the practical contributions of key genetic variations and root traits to crop yield attainment under drought conditions, thereby providing a theoretical foundation for drought-resistant crop breeding. For example, in wheat research, the root system sizes of population materials were assessed by directly measuring the electrical capacitance in the field, and then more developed root systems are screened for their offspring to breed drought-tolerant varieties [16,17]. Rice research shows that introducing quantitative trait loci (QTLs) controlling the root length and thickness from upland rice into rice varieties with strong adaptability but shallow root systems can significantly optimize their root architecture under drought stress and ultimately increase yields in the field [18]. Phenotypic and genetic studies on maize populations reveal that the breeding of modern high-yielding varieties is essentially a process of directional selection and genetic improvement of the “steep root” architecture adapted to dense planting [19]. This study highlights the critical value of root architecture selection in crop breeding.

This review focuses on recent progress in the morphological responses and molecular regulatory mechanisms of root system architecture in major crops (e.g., rice, maize). It covers key root traits (i.e., root length, growth angle, number of adventitious and lateral roots, and root hairs) and their roles in drought stress response. We summarize advances in molecular modules controlling these traits and propose future directions integrating novel technologies to clarify root genetic mechanisms and advance molecular breeding. This review provides a framework for breeding crops with improved root morphology and enhanced drought resilience.

## 2. Root Morphological Traits and Drought Resilience

The root system is the central organ through which crops perceive and respond to drought stress. Crop roots are generally classified into several types including primary roots, adventitious roots, and lateral roots [20,21]. Achieving drought resilience in crops largely depends on the high plasticity of the root system architecture under water deficit. This plasticity is not a passive morphological alteration but an active, adaptive remodeling process that optimizes water acquisition efficiency [22,23]. Generally, drought tolerant root systems enhance their ability to explore and exploit soil moisture through the coordinated adjustment of several key traits [24]. Root length and root angle jointly determine the depth and orientation of root growth, establishing the basis for accessing deeper soil water reserves [25]. The number of adventitious roots and lateral roots, as well as the root hair density, regulate the spatial distribution of the root system and soil–root contact area, directly influencing the water uptake capacity and efficiency [21,26]. Together, these traits form a dynamic framework with inherent functional trade-offs, which collectively mitigate the adverse effects of drought and reduce yield losses [27]. In the following sections, we review recent advances in these key traits and their regulatory mechanisms.

### 2.1. Root Length

When subjected to drought, the elongation of the primary root allows crops to explore deeper soil layers where water may remain available. The proliferation of adventitious roots expands the water acquisition zone in the upper soil profile, thereby enhancing water capture capacity. In addition, the elongation of lateral roots increases the total absorptive surface area of the root system. Overall, crop drought resilience is closely associated with the coordinated elongation of both primary and lateral roots [28]. Drought resistant varieties generally develop deeper root systems. For example, the drought-tolerant rice cultivar ‘Sahabhagidhan’ maintains root growth under water deficit, whereas the sensitive cultivar ‘IR64’ exhibits reduced root length under severe stress [25,29]. The deep rooting trait involves not only an increase in the total root length, but also a preferential allocation of roots along the vertical soil profile. Under drought, rice increases its deep root density, which contributes to early-stage drought tolerance, whereas shallow roots mainly support shoot biomass accumulation [15]. In maize, drought stress during the seedling stage can induce deeper root growth; this “stress imprint” provides a physiological buffer against subsequent drought and mitigates yield loss [30]. Comparative studies in polyploid wheat have shown that historical wheat genotypes had large root systems in the shallow layer, while modern genotypes have more subtle but deeper roots [31]. A new field study states that modern cultivars have improved WUE, but breeding has not strategically enhanced deep root systems to match the needs of water-saving irrigation [32]. It also has to be pointed out that when available water is located in shallow soil, deep root growth becomes an unnecessary investment. Some published field studies have shown that in wet years, the yield of crops with larger root biomass instead decreases [33].

However, under drought conditions, longer root length does not invariably lead to greater drought resilience. For instance, in water-limited environments, species with a low specific root length (SRL)—indicating shorter root length per unit mass, typically associated with thicker and denser roots—often exhibit better performance [34]. Excessively long roots, conversely, incur substantial physiological and ecological costs. These costs are mainly reflected in two aspects. First, a high carbon metabolic burden can inhibit shoot growth. Root growth and maintenance consume large amounts of photoassimilates, estimated to exceed 50% of daily photosynthetic production, thereby directly reducing the allocation of assimilates to seeds and ultimately lowering yield [35]. Second, overly extended root systems can reduce hydraulic efficiency and disturb soil water dynamics. For example, the lesser reduction in grain yield in Tincurrin was associated with slower water extraction by the small root system and slower decline in stomatal conductance, leaf photosynthesis, and transpiration rates [36].

Recent studies on the molecular regulatory mechanisms of crop root systems have revealed that molecular components, which include signal transduction components, transcriptional regulators, and hormone synthesis, metabolism, and signaling components, jointly regulate the growth of root systems [21]. Under drought conditions, root growth in crops is precisely regulated by multi-layered molecular networks. The perception and transduction of drought signals initiate root adaptive responses. In wheat, the Raf-like MAPKKK TaHT1 interacts with the ABA signaling component TaSnRK2.10 to transmit an inhibitory signal of root growth; overexpression of *TaHT1* reduces root length and increases drought sensitivity [37]. Complementing this regulatory layer, small signaling peptides also play key roles. For instance, in wheat, the peptide TaCEP15 interacts with its receptor kinase TaCEPRL, thereby promoting phosphorylation and degradation of the kinase TaSnRK1α and ultimately inhibiting primary root elongation [38].

Signal transduction ultimately converges on the regulation of transcription factors, with the NAC transcription factor family playing a pivotal role in this process. In wheat, overexpression of the root-specifically expressed *TaRNAC1* directly promotes root elongation and biomass accumulation, thereby enhancing drought tolerance [39]. Additionally, R2R3-MYB-type transcription factors exhibit dual functionality. In rice, RRS1 suppresses root development by activating the auxin signaling repressor OsIAA3, while its natural variant allele RRS1(T) attenuates this repression, thereby promoting root length and drought resilience [40]. In maize, the cortex/epidermis-specifically expressed ZmbZIP89 promotes lateral root elongation by activating the peroxidase gene *ZmPRX47*, thereby modulating reactive oxygen species homeostasis [41]. Spatial transcriptomic studies have further elucidated the global transcriptional landscape underlying root adaptation in upland rice, identifying HMGB1 as a core regulator. Its low expression influences hormone signaling pathways such as brassinosteroids (BRs) and coordinates with genes including *CKX4* and *DGL1* to collectively shape a thicker and longer root architecture [42].

Additionally, epigenetic regulation also plays a crucial role in the control of gene expression in root growth [43]. For example, OsRLR4 negatively regulates primary root elongation by precisely regulating the epigenetic modification of *OsAUX1* (an auxin influx carrier gene). In this process, OsRLR4 recruits OsTrx1 (a member of the histone methyltransferase complex) to increase the H3K4me3 level on the promoter of *OsAUX1*. This directly demonstrates that histone modification acts as a “direct regulatory switch” for primary root length [44]. Although this study did not directly describe the effect of drought on epigenetics modification, theoretically, drought signals may recruit or inhibit specific epigenetic modification complexes via certain signaling proteins, reprogramming the epigenetic modification levels of chromatin [45]. Such examples have been verified in wheat research. The histone deacetylase TaHDA8 restricts root elongation under normal conditions by inhibiting the activity of the transcription factor TaAREB3. However, drought stress suppresses the activity of TaHDA8, relieving the inhibition of TaAREB3, which in turn activates downstream target genes such as *TaKOR1*, thereby enhancing root plasticity [46]. Epigenetics is considered as a key mechanism connecting environmental signals to genomic responses, deconstructing genotype–environment (G × E) interactions, and enabling root plasticity. Future research will focus on deciphering the underlying mechanism to elucidate why the same genotype exhibits different root architectures in distinct environments and to identify the inherent differences in their epigenetic regulatory networks.

Transcriptional regulation ultimately converges on a suite of functional genes that directly execute root morphogenesis. Peroxidases play a crucial role in cell wall modification. For example, in maize, *ZmPRX1* enhances the root mechanical strength and elongation capacity by promoting lignin deposition; this gene itself is negatively regulated by the transcription factor ZmWRKY86, forming a coherent regulatory module [47]. Heat shock proteins contribute to proteostasis. Natural variation in the maize gene *ZmHSP20-5* (such as the InDel-1224 in its promoter region) has been identified as a key factor influencing primary and lateral root development; loss of its function impairs root architecture and reduces drought tolerance [48].

The final output of root development is fine-tuned by hormone signaling networks. Under drought, ABA typically restrains root elongation while promoting radial thickening—a drought-avoidance strategy [49]. Concurrently, ABA signaling can also regulate root length through crosstalk with other hormonal pathways. In rice, ABA has biphasic effects on primary root growth through induced auxin biosynthesis. The ABA regulated transcription factor OsbZIP46 specifically promotes the expression of the auxin synthesis gene *OsYUC8*, thereby reducing auxin accumulation at the root tip [49].

In summary, the regulation of root length in crops under drought stress constitutes a synergistic interaction network composed of environmental signals, hormonal signaling pathways, and transcriptional regulation. This network activates or represses specific transcription factors, which in turn drive the extensive reprogramming of downstream functional gene expression. Ultimately, this process orchestrates cellular events such as cell division, elongation, and cell wall modification, thereby achieving precise control over root elongation.

### 2.2. Root Growth Angle

Root growth angle (RGA) is a key trait determining the spatial distribution of root systems in soil, directly influencing the efficiency of water acquisition from different soil layers. Under drought conditions, a smaller root angle (i.e., more vertical downward growth) commonly increases the root depth because more roots penetrate deeper into the soil layers. This root architecture facilitates deeper soil exploration and access to deep soil water reserves, representing an important adaptive trait for drought resilience in crops [50,51]. Field identification of root traits revealed wide variability in root angle among rice and wheat genotypes. Genetic variation in root growth angle has been reported, and this natural variation represents a valuable breeding resource for improving crop productivity under drought stress [52,53]. Introgression of wild-segment fragments into cultivated wheat, combined with field in situ monitoring using electrical impedance tomography, confirmed that the introgression line exhibits a reduced root angle under drought, enabling greater water uptake from deeper soil layers, which in turn sustains higher photosynthetic rates and grain yield [54]. It should also be pointed out that studies in barley have found that the impact of root angle on crop yield requires the comprehensive consideration of genotype, environmental factors such as soil conditions and humidity, and the interactions among them [55]. In climates where crops depend on stored subsoil soil water, a narrow growth angle and larger numbers of seminal roots are beneficial, while in climates with regular in-season rainfall, less narrow growth angles with shallower root system provide better adaptation [56].

**Figure 1:**
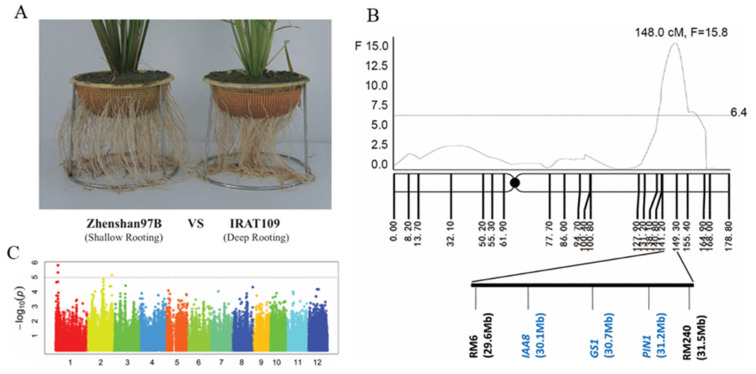
Genetic mapping of rice deep rooting [63]. (**A**) Root phenotype of typical rice with shallow rooting and deep rooting. (**B**) Location of the major deep rooting QTL on chromosome 2. Blue font indicates several known genes located in this region. (**C**) Genome-wide Manhattan plot of the association loci for deep rooting collection.

Root growth angle is primarily controlled by gravitropism. Studies on physiological and molecular mechanisms have revealed that root gravitropism is mainly regulated by the asymmetric distribution of auxin, which was controlled by statoliths (starch-filled amyloplasts) and leads to differential regulation of downstream responsive gene expression. Thus, a molecular network composed of hormonal signals and transcriptional regulators plays a major regulatory role in the process of root growth angle determination [57]. The application of field phenotyping in the study of crop root growth angles has promoted the elucidation of their molecular regulatory mechanisms, and it has been demonstrated that the genetic improvement of key genes can enhance crop yields under field stress conditions [58,59]. In rice, QTL mapping identified *DRO1* (*DEEPER ROOTING 1*) on chromosome 9 of rice as a key gene regulating root growth angle. In rice, *DRO1* is negatively regulated by auxin, and its high expression enhances root gravitropism, promoting deeper root growth. Introduction of *DRO1* into shallow-rooted cultivars significantly improves their yield under drought conditions [58]. Its homolog, *qSOR1*, regulates the rice root angle and yields in saline paddy fields [59]. The function of *DRO1* is conserved across species, as demonstrated by orthologs such as *TaDRO1* in wheat, *ZmDRO1* in maize, *StDRO1* in potato, and *SbDRO1* in sorghum. These genes are specifically expressed in the roots and modulate the root angle in response to stress [60,61,62]. In previous studies, systematic mapping based on linkage analysis and genome-wide association studies (GWAS) identified several major quantitative trait loci (QTLs) that regulate the ratio of deep rooting in rice [63]. Among these, the locus *qRDR-2*, located in the RM6–RM240 interval on chromosome 2, had the most significant effect (Figure 1). This region showed strong selective sweep signals in deep-rooted varieties, indicating its evolutionary relevance for deep rooting and drought adaptation in rice [63]. Further comparative transcriptome analysis revealed widespread differences between deep- and shallow-rooted genotypes in energy metabolism and phytohormone pathways, with deep-rooted tissues exhibiting significantly higher ATP synthesis rates [64]. Recently, a candidate gene within *qRDR-2*, *OsSAUR11*, was identified as an early auxin-responsive gene induced by drought and abscisic acid. *OsSAUR11* is regulated by the transcription factor OsbZIP62 and participates in auxin signaling, thereby promoting an increased proportion of deep roots [65].

Asymmetric auxin distribution serves as the direct driver of root gravitropic bending. This process is primarily mediated by auxin influx and efflux carriers. Following gravity-induced reorientation of the primary root, the intracellular localization of auxin transporters such as PIN3 and PIN7 is rearranged, leading to auxin accumulation in the lower side of the root. This asymmetric distribution subsequently causes differential cell elongation and root tip curvature [66]. In rice, OsPIN1 and OsPIN2 regulate the root angle by mediating polar auxin transport following gravistimulation, and the loss of their function results in impaired gravitropic responses [67]. Similarly, auxin influx carriers such as OsAUX1 participate in regulating root tip auxin dynamics, and mutations in these carriers lead to changed root angles changed root angles through altering nutritropism [68].

The hormonal network centered on abscisic acid (ABA) plays a central role in sensing drought signals and modulating root angle responses. Under drought conditions, ABA levels increase markedly in crop root tips. Research indicates that ABA activates its receptors (e.g., PYL proteins), thereby inhibiting PP2C phosphatase activity and leading to the phosphorylation of SnRK2 kinases. Activated SnRK2.5 further phosphorylates PIN2 proteins, affecting PIN2 vesicular trafficking, auxin transport activity and PIN2-dependent auxin redistribution, thereby compromising root tropic responses and root growth angles [69]. In rice, knockout mutants of the key ABA catabolism gene OsABA8ox2 accumulate higher ABA levels, which induces a more vertical root architecture and enhances drought resilience [70]. Further mechanistic studies reveal that ABA does not directly regulate gravitropism but rather modulates local auxin biosynthesis and distribution. In ABA-deficient mutants, auxin content in the root tip decreases, leading to weakened gravitropic responses and a shallower root phenotype. This defect can be rescued by exogenous auxin application [71]. Together, these findings illustrate how hormonal networks precisely tune root architectural plasticity through sophisticated cross-talk.

Transcription factors also play crucial roles in regulating the root angle. For instance, the transcription factor OsNAC41 can directly bind to and activate the promoter of *RoLe1*—a core gene controlling root angle—and its binding affinity is enhanced by a specific polymorphic site (G-to-T), thereby activating RoLe1 expression. The RoLe1 protein further interacts with the ARF-GTPase-activating protein OsAGAP to jointly regulate root development and angle, providing a transcriptional switch mechanism for root angle regulation [72]. The identification of key transcription factors regulating the root angle and their favorable genetic variations will facilitate the future breeding of new drought-resistant crop varieties with deep root architecture through molecular design breeding.

### 2.3. Number of Adventitious Root and Lateral Root

Root number, which encompasses both adventitious and lateral roots, is a key trait influencing the crop water capture efficiency by expanding the soil–root contact area. Particularly in drought environments where shallow soil moisture is limited, a moderate increase in root number can significantly enhance crop drought resilience [73]. Drought-tolerant cotton cultivars, for example, enlarge their water uptake surface by increasing the length and density of their lateral roots and by optimizing the root tip angles [74]. Using a set of maize introgression lines, root numbers under drought stress were investigated for genetic mapping. A total of 19 significant loci for root number were detected, and 12 were identified in the both medium drought stress and severe drought stress, which would be useful in molecular breeding for drought resilience in maize [75].

Under prolonged drought, excessive root development exacerbates the carbon allocation dilemma. Root tissues incur substantial respiratory costs; thus, a greater number of roots leads to a significant increase in the carbon required for maintenance. Moreover, roots concentrated in dry topsoil exhibit limited water uptake capacity but continuously consume carbon resources, representing a low return investment [76]. Additionally, high densities of lateral and axial roots may increase the total path length and resilience of water flow from the soil to the xylem, and excessive root numbers can reduce the efficiency of internal nutrient redistribution. As drought progresses, plants struggle to flexibly reallocate resources from large yet inefficient root systems, leading to reduced plasticity of the root system architecture [77]. Consequently, under resource limited drought stress, reducing unnecessary root construction costs represents an effective adaptive strategy. In maize, the well-established “Few but Long (FL)” model proposes that genotypes with fewer lateral root branches but more vigorous axial root elongation—compared to the “Many but Short (MS)” type—can lower the root respiratory costs and promote deeper root growth into wetter soil layers. This leads to superior water uptake efficiency, biomass accumulation, and yield under drought conditions [78]. Computational simulations further support the “Steep, Cheap, and Deep” ideotype, in which a reduced axial root number and lateral root branching density enhance biomass production under drought [8].

The polar transport and local accumulation of auxin serve as the driving force for the initiation and development of adventitious roots and lateral root primordia. Auxin transport and signaling components, together with downstream key transcriptional regulators, constitute the main hierarchical regulatory network that influences the number of adventitious roots and lateral roots [28]. For example, loss-of-function of the OsPIN1 family genes (auxin transport proteins) in rice results in a severe reduction in adventitious roots [79]. Overexpression of *TaVAP* in *Arabidopsis* elevates endogenous auxin levels, promoting lateral root proliferation [80]. Cytokinin acts as a negative regulator of adventitious root formation, primarily inhibiting the cell cycle of root meristematic cells [68].

A series of transcription factors act as hub nodes that integrate signals such as drought and hormones, directly regulating the expression of downstream genes involved in root development. In rice, the transcription factor OsWOX11 and its downstream target genes constitute a core regulatory network governing the initiation of adventitious roots [81,82]. OsWOX11 can reduce the cytokinin content in roots by directly regulating the expression of *OsCKX4*, thereby relieving the inhibition of root primordium initiation by the latter [82]. LBD family transcription factors (e.g., CRL1/ARL1) are themselves direct targets of auxin signaling and play a key role in root primordium initiation [81].

Overexpression of *ZmPTF1*, a member of the bHLH transcription factor family in maize, leads to significant increases in lateral root number, seedling root length, and lateral root development under drought stress. Under these conditions, ZmPTF1 binds to the G box cis element in the promoters of NCED family genes, promoting ABA biosynthesis and activating the ABA signaling pathway. Simultaneously, ZmPTF1 interacts with the G box element in the promoters of key regulators involved in stress response or root growth—including CBF4, NAC30, and IAA3—thereby modulating their expression. Collectively, ZmPTF1 enhances drought resilience by promoting root development and activating stress responsive pathways [83].

The transcription factor OsNAC6 positively regulates root number in rice. further study found that OsNAC6 directly binds to the promoter of the nicotinamide synthase gene *OsNAS1* and upregulates its expression, which may increase nicotianamine biosynthesis and promote root development [84].

### 2.4. Root Hairs

Root hairs, as microscopic extensions of epidermal cells, significantly increase the contact area between the roots and soil particles, thereby enhancing the adsorption of capillary water from the soil. They represent an important micromorphological trait for efficient water use under drought conditions [85]. Although no direct effect of root hairs on water uptake was observed in a field trial of maize under drought, they could potentially influence growth dependent on leaf water potential [86]. Barley serves as an optimal crop model for studying root hair traits. Both laboratory and field experiments consistently demonstrate that longer root hairs reduce the decline in water potential at the root–soil interface, help maintain higher transpiration rates, improve plant water status, lower abscisic acid concentrations, and ultimately stabilize yield under drought [87,88]. Loss of function of the *OsRBOHE* gene severely impairs root hair development, which leads to a remarkable reduction in root hair length of the *OsRBOHE* knockout mutant. This weakens root-soil contact and hydraulic connectivity, ultimately reducing drought tolerance in rice [89].

Directly characterizing root hair traits in soil is difficult, but detecting root hairs under hydroponic conditions is effective. Multiple studies have shown that low-phosphorus treatment in hydroponics can reproduce the adaptive responses of plants observed in soil—namely, a significant increase in root hair density and length [90,91,92]. The electric capacitance method is a promising tool for the high-throughput primary screening of root phenotypes. In genetic populations or treatment groups, it enables the quick and easy identification of varieties that may possess more developed root hairs, thereby conferring a larger root absorption surface area [93].

The molecular regulatory mechanism of root hair initiation is a highly conserved and finely regulated process, with its core lying in the determination of root epidermal cell fate regulated by a core transcriptional regulatory module and the polarized growth of root hair cells [94]. Members of the bHLH family of transcription factors play central roles in root hair development and drought response. In rice, OsRSL1, OsRSL2, and OsRHL1; in maize, ZmLRL5; and in wheat, TaRSL2 and TaRSL4, are all involved in regulating root hair elongation under drought conditions. Under drought stress, the expression of these genes is upregulated, promoting root hair elongation and enhancing the water acquisition capacity [95,96,97].

The drought inducible expansin gene *HvEXPB7* is specifically expressed in root hair cells. Silencing *HvEXPB7* significantly represses root hair growth. It is speculated that mechanisms underlying the interaction between the HvEXPB7 protein and cell wall components may exist [98].

Abscisic acid (ABA) acts as a key signaling molecule for root hair cells to perceive drought stress, regulating root hair formation by activating the expression of related genes. In rice, OsSAPK10 promotes root hair elongation via the ABA signaling pathway and influences local auxin distribution by modulating PIN gene expression [99].

These findings reveal that root hair development is programmatically driven by a cascade of key transcription factors, which is subject to multiple regulations by an auxin-centered hormonal network, integrates environmental signals such as drought to coordinate the final length and density of root hairs, and forms adaptive growth responses.

Additionally, as the core interface for plant root–soil interactions, root hairs dominate and mediate the dynamic interplay between the rhizosphere microbiome and soil texture under drought conditions [100]. By secreting rhizodeposits (including sugars, amino acids, phenolics, etc.), root hairs provide abundant carbon sources and a favorable chemical environment for specific plant growth-promoting rhizobacteria (PGPR) and arbuscular mycorrhizal fungi (AMF), thereby “recruiting” beneficial microbial communities to enrich in the rhizosphere. AMF promote the formation and stabilization of root sheaths and improve the soil structure of the rhizosphere microdomain by secreting mucilage, forming hyphal networks, binding soil particles, and creating biological pores, while PGPR and fungi can directly or indirectly promote root hair growth and elongation by producing hormones (e.g., auxin, cytokinin) or secreting signaling molecules, forming a positive feedback loop [101]. Under drought conditions, mechanisms such as root sheath stabilization, water gradient optimization, and phytohormone regulation synergistically enhance crop water acquisition efficiency and drought adaptability, forming a root hair-centered “crop–microbe–soil” synergistic drought-resistant system [102].

## 3. Conclusions and Perspectives

**Figure 2:**
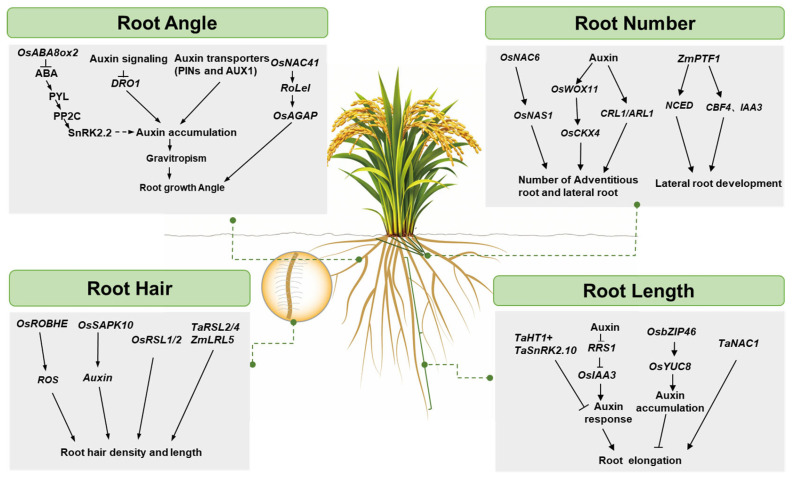
Molecular schematic of the molecular mechanism underlying the regulation of crop root system architecture participating in drought resilience. Molecular modules comprise signaling components, and transcription factors control the root traits including root angle, root length, root number, and root hair.

In summary, root morphological plasticity is a crucial adaptive mechanism for crops to cope with drought stress. Key traits such as root length, root angle, root number, and root hairs collectively shape a root system architecture capable of efficiently sensing and acquiring soil water. The identification of key genes and pivotal nodes within hormonal pathways provides an understanding of the complex genetic and molecular networks regulating root architecture (Figure 2), and will supply a wealth of genetic resources for crop breeding with modified root systems and improved drought resilience through the molecular design and gene editing technique.

However, research in this field still faces numerous challenges: (1) Most studies on molecular mechanisms are conducted during the seedling stage and under controlled environments, and their conclusions require large-scale validation for generalizability across the full growth period under field conditions—especially the critical reproductive growth stage—and in variable natural environments. (2) Complex trade-off relationships exist among root traits, increasing the difficulty of root trait improvement. (3) Different crops exhibit distinct strategies in their root architecture for coping with drought conditions. For example, cereal crops such as maize primarily regulate crown roots and lateral roots, including steeper crown root angles, reduced crown root numbers, and adjusted lateral root density; whereas dicotyledonous crops such as legumes focus on regulating the taproot and basal roots to develop deeper and longer taproots. Secondly, crop roots exhibit different responses under varying drought stress intensities and durations. Under mild to moderate drought, roots display active morphological plasticity—such as inhibiting lateral root formation in dry soil and promoting it on the side in contact with moist soil. Under severe and persistent drought, shallow root branching is inhibited, and root growth is promoted into deeper soil layers to access stable water sources. (4) Standardization of root phenotyping methods remains a major challenge; existing studies struggle to achieve reliable cross-validation via big data analysis. Moreover, the high construction costs of high-throughput root image phenotyping devices and crop cultivation facilities limit the expanded application of root analysis to other crops and large-scale field experiments. (5) Regulatory uncertainties surrounding genetically edited breeding crops restrict the application of genome editing for genetic improvement in staple crops such as rice.

**Figure 3:**
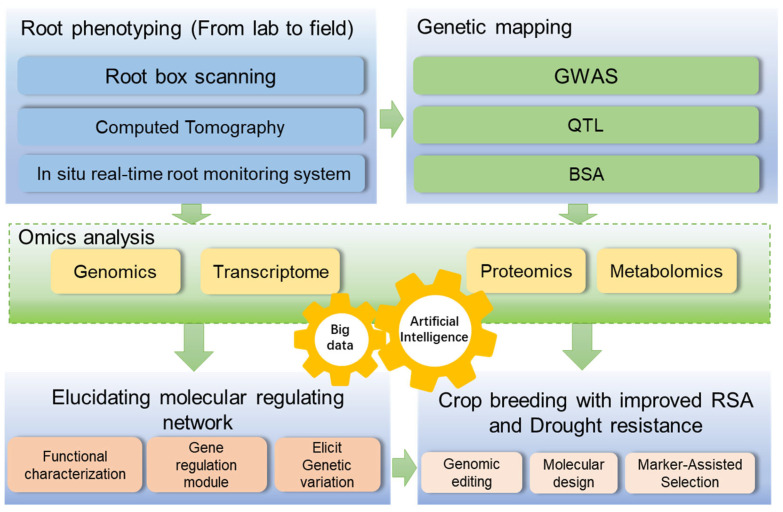
Work flow of future study on the crop root architecture system and breeding. The terms of root phenotyping technologies such as root box scanning and CT imaging were used to precisely capture the root morphological information in the lab, whereas in situ root monitoring systems such as soil core methods and electronical capacitance method remain used in the field test. GWAS, QTL mapping, and BSA analysis will be employed to dissect the genetic basis of root traits. Systems biology approaches (integrating genomics, transcriptomics, proteomics, and metabolomics) will be utilized to deeply explore the molecular regulatory networks underlying root development. Driven by big data from phenotype to omics and artificial intelligence technologies, the mechanistic insights at the molecular genetic level will promote technological innovations in gene editing, molecular design breeding, and marker-assisted selection (MAS) to accelerate the cultivation of superior crop varieties with a modified root system architecture (RSA) and drought resilience.

In future research, the integration of interdisciplinary technologies and the combination of laboratory research with field experiments will accelerate the process of breeding crops for drought-resistant root systems (Figure 3). In the long-term, research must integrate multidisciplinary technologies including root physiology, molecular biology, genetic breeding, soil science, big data, and artificial intelligence. Laboratory research should prioritize solving bottleneck issues related to the improvement of root systems in specific crops under particular field conditions, and establish a matching scheme of the ideal root system with the best cultivation management practices. The soil core method remains the primary technique for precisely quantifying the in situ field root biomass and its spatial distribution [103], whereas the electrical capacitance method is an effective approach for rapidly identifying root differences among varieties in genetic breeding to aid in selecting cultivars with well-developed root systems [93,104]. Field-based root phenotyping observation stations should be established in multiple representative ecological regions, employing the soil core method and capacitance methods as well as other high-throughput, non-destructive, and in situ root phenotyping techniques, combined with UAV remote sensing technology for monitoring aboveground phenotypes. Future research may enable the precise monitoring of root dynamics at the field scale. By utilizing multi-year environmental field phenotyping data and whole-genome sequencing data, genomic selection models for root traits can be constructed, enabling the early and accurate prediction of the root potential of early-stage breeding materials, thereby significantly shortening the breeding cycle.

Multi-omics joint analysis integrating genomics, transcriptomics, metabolomics, and proteomics can systematically dissect the global network of root responses to drought. The maturation of gene editing technologies such as CRISPR/Cas makes it possible to precisely edit key regulatory genes and customize allelic variations. By deeply integrating these technologies with traditional breeding experience, employing multi-gene pyramiding strategies, and targeting specific crops and drought environments, personalized root architecture improvement can be implemented. Furthermore, integrating research on the “root–microbe–soil” interaction will represent a new frontier for comprehensively understanding crop drought resilience and improving field performance. In conclusion, to break the bottleneck in applying basic root system research to practical crop breeding and production, it is essential to deeply consider the complexity of the molecular regulatory mechanisms of root traits and their interactions with the environment, and build a new research ecosystem that is data-intensive, model-driven, deeply integrated with multiple disciplines, and with field performance as its ultimate criterion. With the shift in research approaches and advancements in technology, we will be able to accelerate the design and breeding of a new generation of crop varieties with “ideal-type” drought-resistant root systems, providing fundamental solutions for global food security and sustainable agricultural development.

## Data Availability

No new data were created or analyzed in this study.
